# Hippocampal Pathway Plasticity Is Associated with the Ability to Form Novel Memories in Older Adults

**DOI:** 10.3389/fnagi.2016.00061

**Published:** 2016-03-22

**Authors:** Daria Antonenko, Nadine Külzow, Magda E. Cesarz, Kristina Schindler, Ulrike Grittner, Agnes Flöel

**Affiliations:** ^1^Department of Neurology, NeuroCure Clinical Research Center, Charité University MedicineBerlin, Germany; ^2^Center for Stroke Research, Charité University MedicineBerlin, Germany; ^3^Department for Biostatistics and Clinical Epidemiology, Charité University MedicineBerlin, Germany

**Keywords:** learning, cognitive training, white matter microstructure, diffusion tensor imaging, fornix

## Abstract

White matter deterioration in the aging human brain contributes to cognitive decline. The fornix as main efferent hippocampal pathway is one of the tracts most strongly associated with age-related memory impairment. Its deterioration may predict conversion to Alzheimer’s dementia and its precursors. However, the associations between the ability to form novel memories, fornix microstructure and plasticity in response to training have never been tested. In the present study, 25 healthy older adults (15 women; mean age (SD): 69 (6) years) underwent an object-location training on three consecutive days. Behavioral outcome measures comprised recall performance on the training days, and on 1-day and 1-month follow up assessments. MRI at 3 Tesla was assessed before and after training. Fornix microstructure was determined by fractional anisotropy and mean diffusivity (MD) values from diffusion tensor imaging (DTI). In addition, hippocampal volumes were extracted from high-resolution images; individual hippocampal masks were further aligned to DTI images to determine hippocampal microstructure. Using linear mixed model analysis, we found that the change in fornix FA from pre- to post-training assessment was significantly associated with training success. Neither baseline fornix microstructure nor hippocampal microstructure or volume changes were significantly associated with performance. Further, models including control task performance (auditory verbal learning) and control white matter tract microstructure (uncinate fasciculus and parahippocampal cingulum) did not yield significant associations. Our results confirm that hippocampal pathways respond to short-term cognitive training, and extend previous findings by demonstrating that the magnitude of training-induced structural changes is associated with behavioral success in older adults. This suggests that the amount of fornix plasticity may not only be behaviorally relevant, but also a potential sensitive biomarker for the success of training interventions aimed at improving memory formation in older adults, a hypothesis to be evaluated in future studies.

## Introduction

Age-related changes of brain macro- and microstructure are undisputed major contributors to memory impairments in the course of aging (Grady, 2012). Disrupted integrity in white matter tracts as assessed by diffusion tensor imaging (DTI) can predict cognitive decline in healthy aging and neurodegenerative disease, such as mild cognitive impairment (MCI) and Alzheimer’s disease (AD; Kantarci et al., 2011; Sasson et al., 2013).

Recent studies have shown that cognitive training in the healthy elderly led not only to improved cognitive abilities, but also induced changes in brain structure and functional activation (Belleville and Bherer, 2012; Degen and Schröder, 2014; Chapman et al., 2015). In the study by Chapman et al. (2015), older adults participated in a strategy-based cognitive training for a period of three month with DTI assessments before and after training. Increased white matter microstructural integrity in the left uncinate fasciculus was observed in the training compared to a non-interventional wait-list group. Prior studies have also shown training-induced alterations in white matter microstructure of the healthy aged brain after repeated practice of various memory tasks (Lövdén et al., 2010) and mnemonic techniques (Engvig et al., 2012). Lövdén et al. (2010) demonstrated increased integrity of inter-hemispheric white matter microstructure in the anterior corpus callosum as a consequence of memory and perceptual speed training over a period of 6 month. This finding of an increased white matter integrity was corroborated by Engvig et al. (2012) who moreover demonstrated an association between training-related increases in microstructural integrity and memory improvement.

Memory processes are highly reliant to hippocampal networks and age-related memory decline has been found to be particularly associated with hippocampal atrophy and decreased integrity of forniceal pathways (Metzler-Baddeley et al., 2011; Mielke et al., 2012; Fletcher et al., 2013; Sasson et al., 2013; Douet and Chang, 2014). The fornix carries efferent projections from the hippocampus (Mori and Aggarwal, 2014) and is especially involved in spatial learning and memory (Postans et al., 2014). Its integrity is linked to spatial performance (Postans et al., 2014) as well as to the integrity of the hippocampus (Kantarci, 2014). Furthermore, fornix integrity is decreased in healthy aging, a process accelerated in MCI and AD (Metzler-Baddeley et al., 2012; Pelletier et al., 2013; Zhuang et al., 2013), has a particular role in predicting episodic memory performance (for a review, see Douet and Chang, 2014) and may mediate hippocampal atrophy (Pelletier et al., 2013). It is among the earliest abnormalities in cognitively normal individuals at risk for AD (Douaud et al., 2013), may predict the conversion to MCI and AD (Douaud et al., 2013; Kantarci, 2014) and thus can serve as an imaging biomarker for cognitive decline in preclinical stages of AD (Mielke et al., 2012; Zhuang et al., 2013; Douet and Chang, 2014).

Previously, fornix micro- and macrostructure have been associated with visual scene discrimination in young adults (Postans et al., 2014). Moreover, a 2-h short-term training in a car-racing task had the potential to induce microstructural changes in the hippocampus (Sagi et al., 2012). These changes were associated with both behavioral improvement in the task as well as with white matter microstructure of the fornix (Hofstetter et al., 2013). However, the predictive quality of microstructural changes in the fornix in older adults, as induced by cognitive training, on training success is still unknown.

In the present study, our aim was to investigate short-term fornix plasticity in older adults and its association to training success. Therefore, healthy older adults were administered an object-location training task (Flöel et al., 2012; Külzow et al., 2014) on three consecutive days. Task performance was assessed on each training day, and on 1-day and 1-month follow-up assessments. Before and after training, participants underwent structural scanning at 3 Tesla for DTI to determine baseline values as well as changes in fornix microstructure as determined by fractional anisotropy (FA) and mean diffusivity (MD) values. Given the particular role of the forniceal pathway, we hypothesized a specific role of its plasticity for training success. For comparison, we also assessed individual hippocampal volume from high-resolution brain images (T1) and hippocampal MD from DTI.

## Materials and Methods

### Participants and Study Design

Twenty-five healthy (15 women; mean age (SD): 69 (6) years, range: 56–78) older adults participated in the study. All were native German speakers, were right-handed, and had no history of neurological or psychiatric disorders. All underwent neuropsychological testing prior to study inclusion in order to assure normal cognitive functioning (CERAD-Plus, memoryclinic.ch). The study was approved by the ethics committee of the Charité University Medicine and conducted in accordance with the Helsinki Declaration. Written informed consent was obtained from all participants prior to participation.

**Figure 1:**
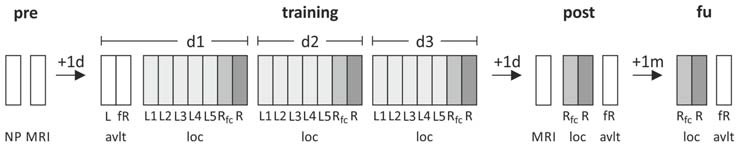
**Study design.** Participants underwent a training of the object-location paradigm in five blocks followed by free recall and recognition trials on three consecutive days. As a control task, Rey Auditory Verbal Learning Test (AVLT) was administered. Prior to and 1 day after training a structural MRI, including high-resolution T1 and diffusion-weighed images, was assessed. Post-assessment included recall and recognition testing which was repeated in a 1-month follow-up assessment. NP, neuropsychological testing; MRI, magnetic resonance imaging; loc, object-location paradigm; avlt, auditory verbal learning task; L, learning; Rfc, 3-alternative-forced-choice recall task (AFC); R, recognition; fR, free recall.

All participants completed a training of the object-location paradigm on three consecutive days followed by immediate cued recall and recognition testing. Recall and recognition performance were again measured 1 day after training as well as in a 1-month follow-up assessment. In order to assess structural training-induced alterations in memory-related neuronal circuits, MRI was conducted 1 day before and after training. As a control task for episodic memory, performance on the German version of the Rey Auditory Verbal Learning Test (AVLT; Helmstaedter et al., 2001) was assessed on each time point (training, post and follow-up assessment; see Witte et al. (2014b) for a detailed description of the task; Figure 1).

### Object-Location Learning Paradigm

For a detailed description of and rationale behind the object-location paradigm, see (Flöel et al., 2012; Külzow et al., 2014). In brief, participants had to learn correct positions of buildings on a street map. Buildings occurred on “correct” and “incorrect” locations, the “correct” pairings of stimulus and location (total of 30 buildings) presented ten times more frequently over the course of five learning blocks. Subjects responded to 120 stimulus-location pairing in each block (60 “correct”; 60 “incorrect”) by button press. Response interval was restricted to stimulus presentation (3 s). Online feedback was not provided. Immediately after five learning blocks, learning success was tested with a cued recall and a recognition task. Here, half of the object-location associations (i.e., 15) was tested in a 3-alternative-forced-choice recall task (AFC) while the other half was tested in simple yes/no item recognition task. During AFC, participants had to indicate the “correct” positions of a building which was presented above the street map, three possible positions (one correct, two incorrect) appeared as numbers. During recognition, stimulus presentation was identical to learning blocks and participants had to indicate whether the object-location pair was correct, timing was self-paced. Recall and recognition tasks were also administered 1 day after training as well as in a 1-month follow-up assessment. Performance was measured by the percentage of correct responses. Recall performance was used as main outcome variable (Flöel et al., 2012). Two different versions of the task were implemented that included different stimuli (*n* = 12 performed version A). The task was administered using the software Presentation^1^.

### MRI Acquisition

MRI was conducted prior to and after training with a 3T Siemens Trio MR-System using a 12-channel head coil at the Berlin Center for Advanced Neuroimaging. In each session, a 3D structural scanning protocol using high-resolution T1-weighted magnetization prepared rapid gradient echo (MPRAGE) imaging (TR = 1900 ms, TE = 2.52 ms, 192 sagittal slices, voxel size = 1.0 × 1.0 × 1.0 mm^3^, flip angle = 9°), and a diffusion-weighted spin-echo echo-planar imaging (EPI) sequence (TR = 7500 ms, TE = 86 ms, 61 axial slices, voxel size = 2.3 × 2.3 × 2.3 mm^3^; 64 directions, b-value of 1000 s/mm^2^, 10 b0) were measured. An additional fluid attenuated inversion recovery (FLAIR) sequence was acquired in order to exclude structural abnormalities.

### MRI Analysis

Analysis of the individual T1 and diffusion-weighted images were performed with FSL^2^ and FreeSurfer^3^. Preprocessing of diffusion weighted images included eddy current and motion correction, extraction of non-brain tissue and fitting of a tensor model to obtain individual 3D maps of FA and MD (Behrens et al., 2003). Images at all processing stages were visually inspected to exclude erroneous brain extraction, registration, and segmentation.

#### Fornix Microstructure

**Figure 2:**
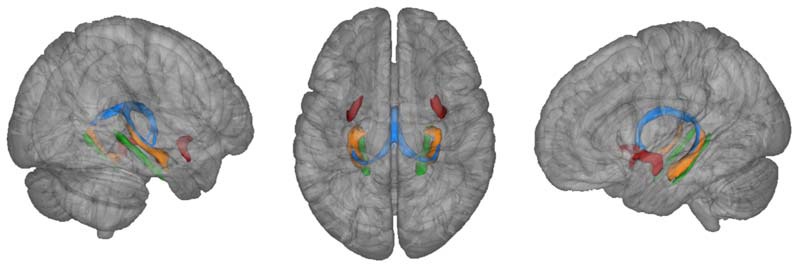
**Fornix mask (blue) and uncinate fasciculus (red) extracted from Juelich atlas (thresholded at 50%) and parahippocampal mask (green) extracted from JHU atlas (thresholded at 20%) as implemented in FSLView.** For illustration purposes, atlas-based hippocampi are also depicted (orange).

An atlas-based approach was used in order to derive microstructure of the fornix. Therefore, the probabilistic fornix mask was extracted from the Juelich histology atlas implemented in FSLView and thresholded to contain only voxels with a minimum probability of 0.5 of inclusion in the mask (Figure 2; Antonenko et al., 2013). Thresholded masks were binarized and transformed into individual diffusion spaces using affine registration (Jenkinson and Smith, 2001). Individual fornix masks were then used to extract MD and FA values.

The same procedure was applied to extract MD and FA values from the uncinate fasciculus and parahippocampal cingulum that served as a control white matter region (mask for the letter derived from the JHU white-matter atlas, thresholded at a probability of 0.2; Metzler-Baddeley et al., 2011). Masks for left and right control white matter regions were averaged for statistical analysis.

#### Hippocampal Segmentation

FMRIB’s Integrated Registration and Segmentation Tool (FIRST) was applied to high-resolution T1-weighted images to perform a model-based segmentation of the left and right hippocampus (Patenaude et al., 2011; Kerti et al., 2013; Witte et al., 2014b). Individual hippocampal volumes were adjusted for total intracranial volume (den Heijer et al., 2012; Kerti et al., 2013; Witte et al., 2014b). T1-weighted images were coregistered to the b0 DTI images using rigid body transformations and these registrations were used to transform individual masks of left and right hippocampus to individual MD maps in order to extract individual hippocampal MD values (Kerti et al., 2013; Witte et al., 2014b). Since preliminary analysis yielded similar results considering left and right hippocampal volumes as well as MD values, they were averaged for statistical analysis (Pelletier et al., 2013).

### Statistical Analysis

Statistical analyses were conducted with IBM SPSS 22^4^. Paired *t*-tests of changes in fornix MD and FA as well as hippocampal MD and volume before and after training were conducted. Linear mixed models (random intercept models) were calculated for the outcome variable task performance (Meinzer et al., 2014) where the time points during learning or recall, respectively, were level-one units nested in the different individuals who were level-two units. In order to describe the learning curve on the training days 1, 2, and 3, linear and quadratic terms for time points (centered) were entered into the model. The main linear mixed model for the variable recall performance tested for the effects of fornix FA change from pre to post training assessment. This model was adjusted for baseline fornix FA, for all five assessments, and for age. Additional models tested characteristics associated with fornix MD, hippocampal volume and hippocampal MD. As control, we further tested associations with a control task (AVLT) as well as a control white matter tract (uncinate fasciculus, parahippocampal cingulum).

In order to infer the amount of explained variance by our model and fornix FA change, we computed marginal and conditional R^2^ measures as proposed by (Nakagawa and Schielzeth, 2013; Johnson, 2014). The marginal R^2^ is a measure of variance explanation that is attributable to the fixed effects of the model. The conditional R^2^ includes additionally variance that is explained by the random effects.

A two-sided significance level of α = 0.05 was used. No adjustment for multiple testing was applied.

## Results

### Training Performance

**Figure 3:**
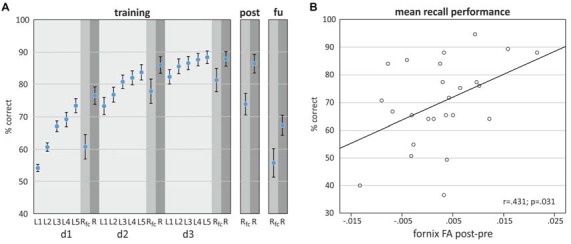
**(A)** Object-location task performance on training days d1, d2, and d3, 1 day after training (post) and 1-month follow up (fu) assessment. **(B)** Scatterplot showing the relationship between the difference in fornix FA (post-pre) and mean recall performance (±SEM), derived by averaging performance over all time points.

Task performance improved over learning blocks and training days (Figure 3A). Improvement was significant with regard to the 15 time points in 3 days in a curvilinear convex manner indicated by a significant linear increase (β for time point (centered, linear) = 2.20, SE = 0.06, *p* < 0.001) and an additional significant coefficient for the square of time points (β for time points (squared) = −0.16, SE = 0.02, *p* < 0.001; data not shown, linear mixed model, *N* = 25 participants/375 measures).

### Association of Fornix Microstructure Alterations with Training Performance

Comparison of fornix FA and MD between post and pre assessment indicated differences in the direction of a higher fornix FA and a lower MD after the training, however only the MD difference reached statistical significance (Table 1). A linear mixed model revealed that the difference of fornix FA from pre- to post-training assessment was significantly associated with recall performance, adjusted for baseline fornix FA, age and assessment day (Table 2). Thus, the higher the increase in fornix FA from pre to post measurement, the better the recall performance in the object-location task (Figure 3B depicts a scatterplot of fornix FA difference and mean recall performance; here, Pearson’s correlation coefficient is indicated). Including recall performance at follow-up assessment in the model, fornix FA increase from pre to post measurement was still associated with superior performance (*p* = 0.057, data not shown). Change in fornix MD and baseline FA and MD levels were not significantly associated with recall performance (Table 2).

**Table 1:** **Fornix microstructure and hippocampal microstructure and volume**.

	pre mean (SD)	post mean (SD)	*p*
**Fornix**
FA	0.232 (0.029)	0.235 (0.028)	0.114
MD (10^−3^ m^2^/s)	1.969 (0.022)	1.949 (0.024)	0.036
**Hippocampus**
MD (10^−3^ m^2^/s)	1.111 (0.008)	1.115 (0.009)	0.669
Vol (in 10^3^ mm^3^)	3.863 (0.423)	3.845 (0.382)	0.505

*FA, fractional anisotropy; MD, mean diffusivity; Vol, volume. N = 25 subjects*.

**Table 2:** **Linear mixed effect models (random intercept models) for recall performance (adjusted for time point and age (centered))**.

	**Fornix**				**Hippocampus**			
	**FA**		**MD**		**MD**		**Vol**	
	β (SE)	*p*	β (SE)	*p*	β (SE)	*p*	β (SE)	*p*
**Fixed effects**
Intercept	40.4 (24.1)	0.108	56.0 (29.5)	0.071	54.9 (43.0)	0.216	75.1 (33.0)	0.034
Baseline (pre)	54.3 (101.7)	0.599	−1* (14.7)	0.960	0.2* (38.5)	0.995	−0.01 (0.01)	0.547
Change (post-pre)	792.4 (345.8)	0.033	−23.7* (67.3)	0.728	23.3* (57.2)	0.688	0.01 (0.02)	0.758
**Random effects**
Variance between subjects (Intercept)	138.5 (52.3)	0.008	179.5 (64.8)	0.006	179.5 (64.7)	0.006	177.0 (64.0)	0.006
Residual variance	142.5 (20.7)	<0.001	142.5 (20.7)	<0.001	142.4 (20.7)	<0.001	142.5 (20.7)	<0.001
Marginal R^2^	0.41		0.33		0.33		0.34
Conditional R^2^	0.70		0.70		0.70		0.70

FA, fractional anisotropy; MD, mean diffusivity. *N* = 25 subjects/124 measures. *in 10^3^.

Variance explanation of recall performance due to FA fornix change was 7% (marginal R^2^ in model with difference of Fornix FA as covariate: 0.41, Table 2, marginal R^2^ without difference of Fornix FA in the model: 0.34, data not shown).

Differences between pre and post assessments were not significant for FA and MD extracted from control white matter tracts (uncinate fasciculus: *p* = 0.47 and *p* = 0.18; parahippocampal cingulum: *p* = 0.19 and *p* = 0.81) and were also not significantly associated with recall performance (uncinate fasciculus: β = 390.8, SE = 242.4, *p* = 0.122; parahippocampal cingulum: β = −134.2, SE = 173.4, *p* = 0.448). Fornix FA change was not significantly associated with control task performance (β = 75.3, SE = 58.6, *p* = 0.214).

Comparison of hippocampal MD and volume between pre and post assessment did not show significant differences between post and pre measurements (Table 1). Change in hippocampal MD and volume as well as baseline values were not significantly associated with recall performance (Table 2).

## Discussion

In the present study, we implemented a short-term object-location training in older adults and assessed hippocampal pathway microstructure using DTI before and after training. The results showed an association between increase in FA in the fornix from pre- to post-training with behavioral training success over training days, 1-day as well as 1-month follow-up assessment.

DTI-derived indices such as MD (i.e., molecular diffusion rate) and FA (i.e., directional preference of diffusion) are widely used to quantify the integrity of tissue microstructure and fiber organization (Johansen-Berg and Behrens, 2009; Soares et al., 2013), with lower MD and higher FA indicating superior white matter microstructure in the majority of reports (Kantarci et al., 2011; Acosta-Cabronero and Nestor, 2014). Inter-individual variability in these indices has been associated with behavioral performance variability in various cognitive tasks (Kantarci et al., 2011; Antonenko et al., 2013; Sasson et al., 2013). Moreover, these DTI metrics can be applied to study brain plasticity *in vivo* (Scholz et al., 2009; Hofstetter et al., 2013) as their alterations are linked to electrophysiological phenomena such as long-term potentiation (LTP; Matsuzaki et al., 2004; Sagi et al., 2012). Cellular microstructural alterations (e.g., density, myelin etc.), indicative of LTP induction, may thus be evident in DTI metrics. In fact, regional MD decrease correlated with concomitant brain-derived neurotrophic factor (BDNF) increase that is a marker of LTP (Sagi et al., 2012). Neuronal changes as quantifiable by DTI metrics were induced by long-term cognitive interventions (Scholz et al., 2009). First evidence that DTI metrics are also susceptible to interventions on a short timescale was provided by Assaf and his colleagues who demonstrated that short-term learning can induce structural DTI-based plasticity (Sagi et al., 2012; Hofstetter et al., 2013).

Our study complements these recent observations of short-term white matter plasticity and its relationship to training success (Sagi et al., 2012; Hofstetter et al., 2013). The authors showed microstructural changes in hippocampal structures after a car-racing-task training of only 2 h (Sagi et al., 2012) and a correlation of training-induced hippocampal and forniceal changes with behavioral improvement in young adults (Hofstetter et al., 2013). In the present study, we did not observe alterations in MD or volume of the hippocampus from pre- to post assessments, nor associations of these brain parameters with performance. In the fornix, however, we found a decrease in MD and a corresponding increase in FA after training. The microstructural change from pre- to post-training assessment was more pronounced in the fornix MD (similar to the study of Hofstetter et al., 2013) with the fornix FA change not reaching statistical significance in a paired *t*-test. Note that despite FA increases in the majority of subjects, some participants showed FA decreases over the short period of time. This finding is consistent with other studies (Sagi et al., 2012; Hofstetter et al., 2013). However, interpretation of these changes remains difficult and has to be scrutinized in future studies.

The association of fornix FA alterations with recall performance over multiple time points in our study supports a role of the fornix for learning. Linear mixed models that were adjusted for age, baseline microstructure and assessment time revealed an association between fornix plasticity and performance. Analysis of the amount of explained variance revealed that fornix microstructural plasticity may be a significant contributor to inter-individual variability in training success. Given that the difference in FA from pre- to post-training, but not fornix FA at baseline (pre), was linked to training success, we argue that the magnitude of hippocampal pathway plasticity, rather than its integrity *per se*, may be predictive of learning capability (cf. Sagi et al., 2012). Other studies with older adults have shown specific associations of fornix integrity and age-related cognitive decline (Metzler-Baddeley et al., 2011; Pelletier et al., 2013); however, our study is the first that linked training-induced structural plasticity and training success in aging.

Statistical models of hippocampal microstructure and volume did not reveal a relationship with performance. This observation may indicate a crucial role of hippocampal pathways, rather than the hippocampus itself, for learning plasticity in older adults. This assumption has also been supported by recent investigations suggesting that loss of fornix connections mediates hippocampal atrophy (Pelletier et al., 2013), and fornix microstructural degradation precedes hippocampal atrophy (Zhuang et al., 2013). Similarly, microstructural abnormalities in the hippocampal system seem to occur even before clinical progression to AD and its precursors (Mielke et al., 2012). Our results thus lend support to the hypothesis that magnitude of hippocampal pathway plasticity in older adults may be decisive for learning and memory formation.

The rapidly rising number of older adults leads to a strong increase in age-associated diseases, including MCI and AD (Petersen et al., 2009). There is high public health interest for early identification of healthy individuals at risk for cognitive decline, to allow for targeted interventions in the preclinical stage, including cognitive training. Given that hippocampal pathway alterations result at least in part from the aging process (Pelletier et al., 2013), it is important to understand the mechanisms underlying these changes, and their associations with behavioral parameters. Our study provides first evidence that hippocampal pathway plasticity may be a sensitive biomarker for success of behavioral interventions aimed at improving memory formation in older adults. Future studies have to evaluate: (i) if the plasticity induced over short time-scales may also predict long-term training success of trained items, and possibly even transfer of training effects to activities of daily life. Moreover, it should be evaluated (ii) if interventional strategies aiming to counteract early structural abnormalities in brain white matter (e.g., supplementation with high dose fatty acids (Witte et al., 2014a)) beneficially modulate both training success and structural brain plasticity, and may therefore open novel therapeutic strategies particularly for individuals with no or only limited training-induced plasticity in the naïve state.

Several limitations should be considered when interpreting our findings. First, fornix FA changes from pre- to post-training assessment were not statistically significant in a paired *t*-test. However, both MD and FA changes complemented each other in the direction of an improved white matter microstructure following training. Moreover, given the short time period, we would only expect small and not extreme alterations in regional microstructure and thus, changes may not be evident in statistical comparisons. Potential reasons may be increased noise (Hofstetter et al., 2013), small sample size, and also gender effects (also discussed by Sagi et al. (2012); who observed more pronounced MD decreases in males; Lisofsky et al., 2015). Second, we focused on DTI metrics 1-day before and after training with no repetition of DTI assessment in a 1-month follow-up. However, we demonstrated that the magnitude of microstructure changes, as assessed directly after training, was also associated with performance at follow-up. Future studies using additional DTI measurements should now determine if structural alterations are persistent over time. Third, additional control groups that undergo no or a different intervention should corroborate the specificity of the present findings. However, we analyzed the effects of fornix FA changes on a control task as well as changes in control white matter tracts on training task performance which did not yield significant associations and thus suggests regional specificity of the findings.

In sum, our study suggests that the healthy aging brain not only retains the potential of dynamic white matter microstructure alterations but that this plasticity may be relevant for the ability to form novel memories. Specifically, fornix plasticity after brief trainings may be a sensitive biomarker for success of long-term behavioral interventions aimed at improving memory formation in older adults, a hypothesis to be evaluated in future studies. Furthermore, in individuals with no or limited plasticity after short-term training, interventions that have previously been shown to improve white matter microstructure, like omega-3 fatty acid supplementation (Witte et al., 2014a), may be evaluated as training-adjuvant means.

## Author Contributions

DA, NK, and AF designed research; DA, NK, and MEC performed research; DA, NK, MEC, KS and UG analyzed data; KS and UG provided tools for statistical analysis; DA and AF wrote the article; DA, NK, MEC, KS, UG and AF commented and approved the final version of the manuscript.

## Conflict of Interest Statement

The authors declare that the research was conducted in the absence of any commercial or financial relationships that could be construed as a potential conflict of interest.

## Footnotes

^1^https://www.neurobs.com

^2^http://www.fmrib.ox.ac.uk/fsl

^3^http://surfer.nmr.mgh.harvard.edu/

^4^http://www.ibm.com/software/de/analytics/spss/
